# Analyses and Research on a Model for Effective Thermal Conductivity of Laser-Clad Composite Materials

**DOI:** 10.3390/ma16237360

**Published:** 2023-11-27

**Authors:** Yuedan Li, Chaosen Lin, Bryan Gilbert Murengami, Cuiyong Tang, Xueyong Chen

**Affiliations:** 1 College of Mechanical and Electrical Engineering, Fujian Agriculture and Forestry University, Fuzhou 350001, China; 2 College of Mechanical and Electronic Engineering, Northwest A&F University, Yangling, Xianyang 712100, China

**Keywords:** mathematical modeling, thermal conductivity, heat transfer material, laser cladding

## Abstract

Composite materials prepared via laser cladding technology are widely used in die production and other fields. When a composite material is used for heat dissipation and heat transfer, thermal conductivity becomes an important parameter. However, obtaining effective thermal conductivity of composite materials prepared via laser cladding under different parameters requires a large number of samples and experiments. In order to improve the research efficiency of thermal conductivity of composite materials, a mathematical model of Cu/Ni composite materials was established to study the influence of cladding-layer parameters on the effective thermal conductivity of composite materials. The comparison between the model and the experiment shows that the model’s accuracy is 86.7%, and the error is due to the increase in thermal conductivity caused by the alloying of the joint, so the overall effective thermal conductivity deviation is small. This study provides a mathematical model method for studying the thermodynamic properties of laser cladding materials. It provides theoretical and practical guidance for subsequent research on the thermodynamic properties of materials during die production.

## 1. Introduction

The utilization of multiple material structures to create composite materials with diverse material properties has proven to be a valuable approach for enhancing material characteristics and tailoring them to specific requirements [1]. These solutions offer numerous advantages and have found widespread applications in the aerospace industry [2]. Laser cladding is a technology in which the metal surface is melted by using a high-energy laser beam to form a molten pool, and the metal powder is deposited on the metal surface under the action of laser energy. The resulting clad layer exhibits unique properties influenced by various parameters, including laser power, scanning speed, and cladding process parameters [3,4,5,6,7,8].

Laser cladding can produce high-hardness and wear-resistant coatings with long-term stability [9,10,11]. Research shows that laser cladding technology can improve the service of coatings in high-temperature and other harsh environments [12]. This method has great value and significance for manufacturing reinforced coatings on the surface of high-thermal-conductivity materials.

A significant advantage of laser cladding lies in its ability to rapidly produce cladding layers with varying thicknesses and compositions. However, determining the effective thermal conductivity under different material thicknesses necessitates a substantial number of samples and experiments [13]. While past research on laser cladding has primarily focused on enhancing mechanical and wear properties, the evaluation and prediction of thermal conductivity in laser-clad composites has received relatively limited attention [14]. Reliable prediction of effective thermal conductivity is an important aspect of material design strategy for key engineering applications [15,16]. Therefore, establishing a thermal resistance digital model of composites fabricated via laser cladding will become a preliminary but key step for the rapid selection of cladding-layer materials and process parameters to achieve efficient composite design in the future.

The existing literature shows that the thermal conductivity models of composites can be roughly divided into three types: empirical models, finite element models, and theoretical models. Empirical models are mostly applied to mixed materials that cannot be combined into compounds. Yang et al. [17] prepared different test blocks of rubber sand composites through different proportions and particle sizes for multiple series of thermal conductivity tests, normalized them, and established a prediction model. The prediction model established by this method had good accuracy. Finite element models are mostly used in composites with complex three-dimensional structure models. Yan et al. [18] built a model of uranium dioxide (UO_2_)/silicon carbide (SIC) composite by connecting the grains of the two materials with different proportions randomly generated by a computer, and calculated and analyzed its effective thermal conductivity using the finite element method (FEM). The model managed to reduce the design speed and costs. Most of the theoretical models are applied to three-dimensional structural composites with certain rules. Fang He et al. [16] proposed a thermal conductivity model of aerogel-filled thermal insulation composites. According to the dispersion of aerogel particles, the discrete model of aerogel composites was established to generate gaps and holes, and the corresponding thermal conductivity was derived. In a study by Witold weglewski et al. [19], the relationship between a coating material and its thermal conductivity was modeled using the finite element method (FEM) and variational asymptotic method (VAM). Using micro-CT modeling technology, the microstructure of the composite was used to generate the finite element mesh model, and the thermal conductivity of the composite was calculated according to the model results.

Compared with theoretical models, it can be seen that empirical models and finite element models have strong generality for the prediction of the thermal conductivity of composite materials. The disadvantage is that when used in similar composite materials, these models need to be reestablished according to the material properties. A finite element method model is slow to establish, and the modeling time is long; the universality of empirical formula is poor, and repeated tests are needed for different materials.

Considering the above problems, laser-clad composites have structural solid similarities. This project aims to develop a theoretical model to rapidly predict the effective thermal conductivity of copper/nickel gradient materials. The model considers the thermal conductivity of dissimilar materials and the cladding layer’s thickness. The effects of different parameters on the thermal conductivity of composites were verified by means of model prediction and experimental comparison. Using the established general model, the influence of key parameters on the effective thermal conductivity of composites prepared via laser cladding was studied, which has theoretical significance for guiding the parameter design of laser-clad composites, improving the design speed, and reducing the development cost.

## 2. Physical Model of Composites

**Figure 1:**
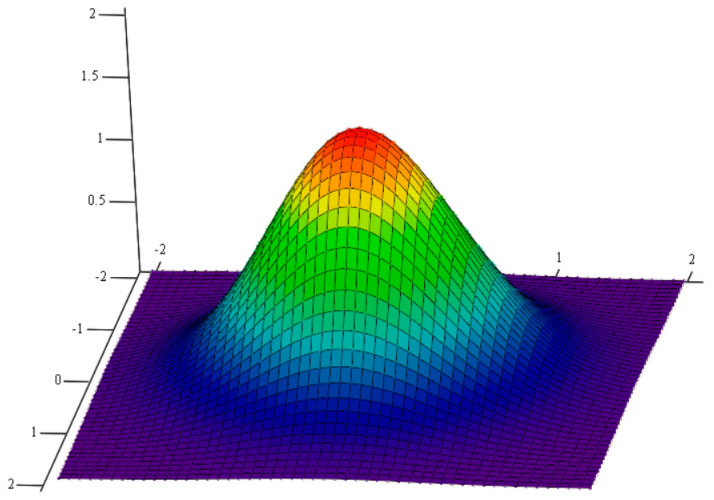
Gaussian-distribution diagram.

The structure of laser-clad composite materials has a certain degree of regularity. To accurately predict the effective thermal conductivity of composite materials, their structure is examined. The light source energy of laser cladding is Gaussian distributed [20], which is explained by the Gaussian heat source model shown in Figure 1 [21]. In this model, the heat source energy is distributed in a circular plane according to a Gaussian function, and calculated by using Equation (1):

(1) $$q\left(x,y\right)=\frac{\eta Q}{2\pi r^{2}}\exp(\frac{x^{2}+y^{2}}{2r^{2}})$$

where $q\left(x,y\right)$ is the density of heat flux at the coordinate point; η is the rate of laser energy absorption by the surface of the material; Q is the laser light source’s output; and r is the radius length of the laser source.

**Figure 2:**
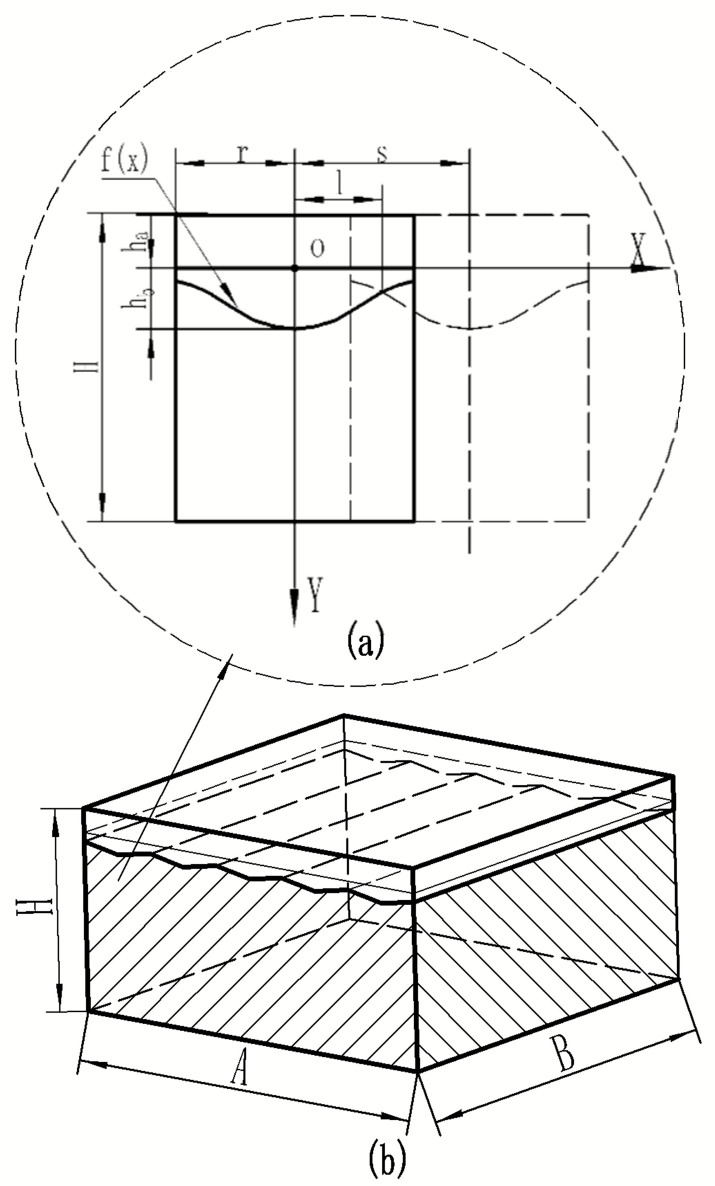
Physical model of composites. (**a**) Composite material unit model. (**b**) The overall distribution model of composite materials.

The surface of metal materials absorbs energy to melt and combine with the metal in a short period of time. Under the action of a Gaussian-distributed laser light source, the surface area of the joint after laser cladding can be approximated as a Gaussian distribution. As shown in Figure 2, the periodic structure generated plays a crucial role in the heat conduction of composite materials. A periodic composite material structure model was proposed for the convenience of calculation to predict the effective thermal conductivity of composite materials.

In this figure, A represents the length of the composite material; B represents its width; H represents the composite element model’s total height; S represents the distance between adjacent laser claddings; R is the radius of the laser’s light source cladding; $h_{a}$ is the thickness of the cladding layer’s material; $h_{b}$ is the height of the first laser cladding layer; and (b) is the composite element model.

The uppermost layer comprises the surface cladding material, while the intermediate layer is the bonding layer. The function of the cladding material’s connection to the substrate is the Gaussian distribution of the formula $f\left(x\right)=h_{b}\exp\left(-x^{2}/{\left(0.5r\right)}^{2}\right)$. The overall distribution model of composite materials is (b).

## 3. Effective Thermal Conductivity Model of Composites

A thermal conductivity model of composite materials prepared via laser cladding is investigated in this paper. The principal research factors are defined as the cladding-layer characteristics, process parameters, and structural characteristics. Considering the model’s precision and the need to reduce the calculation’s complexity, the following parameters are simplified:

1. The interior of metal is uniform and devoid of cavities.
2. During laser cladding, the alloying of two dissimilar alloy materials is disregarded.
3. The material’s surface is polished and evaluated after preparation; therefore, the oxide layer is not considered, and the thermal resistance of the interface is not accounted for in the calculation.
4. The element model is a cross-section perpendicular to the direction of laser cladding.

### 3.1. Model of Element

**Figure 3:**
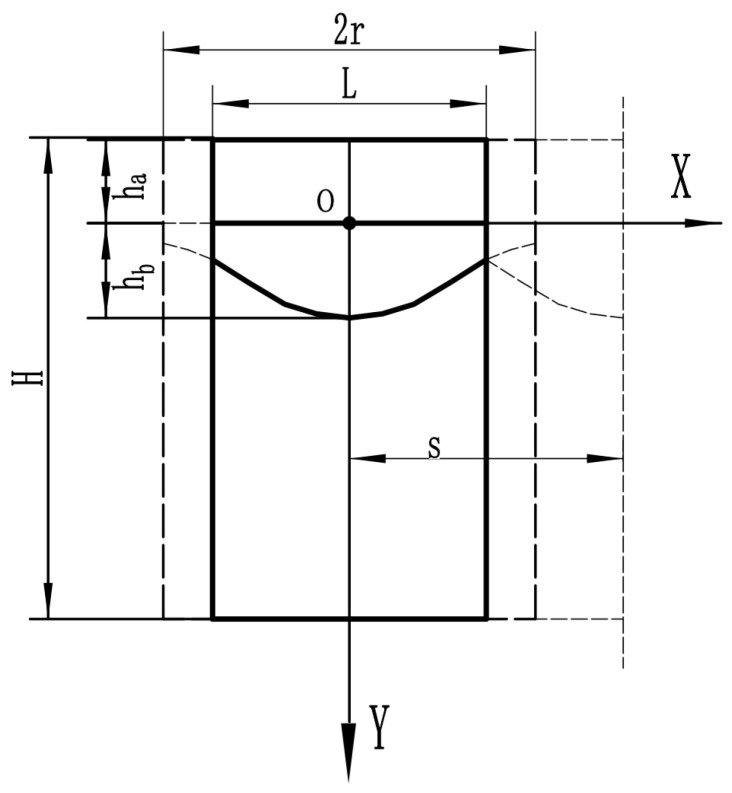
Composite material unit model.

Figure 3 depicts the composite element model clad in laser; s is the distance between two element models, and they overlap. The composite material consists of three distinct components. The first component is the cladding layer, the second is the cladding layer’s bonding position, and the third is the base material. Under the same material, it is presumed that temperature is linearly distributed in a single effective conduction direction [16]. The effective actual cell width is determined as follows:

(2) L=2l=2r−s

where r is the radius of the laser cladding, s is the distance between adjacent laser claddings, and l is half the effective unit length. At the intersection, according to the previously established boundary approximation curve f(x),

(3) $$f\left(x\right)=h_{b}\cdot e^{\frac{-x^{2}}{{\left(0.5r\right)}^{2}}}$$

**Figure 4:**
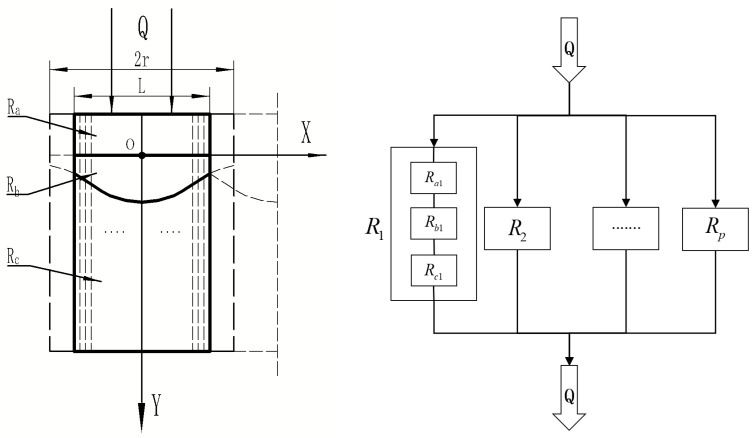
Unit model is divided into multiple parallel thermal resistance regions.

Since the model’s contact surface is a Gaussian function, the joint surfaces are nonparallel. To accomplish the purpose of calculating the equivalent thermal calculation, it is subdivided vertically into a number of small ideal elements. When the difference is negligible, it may be considered parallel. Figure 4 depicts this. In this investigation, it is divided symmetrically along the Y axis into two regions that are considered to have parallel thermal resistance. The final result will incline toward a value as the regional mean fraction P increases.

According to the thermal resistance calculation formula, the equivalent thermal resistance of the unit model is calculated as follows:

(4) $$\frac{1}{R_{o}}=2\cdot \left(\frac{1}{R_{1}}+\frac{1}{R_{2}}+\ldots \ldots +\frac{1}{R_{p-1}}+\frac{1}{R_{p}}\right)$$

**Figure 5:**
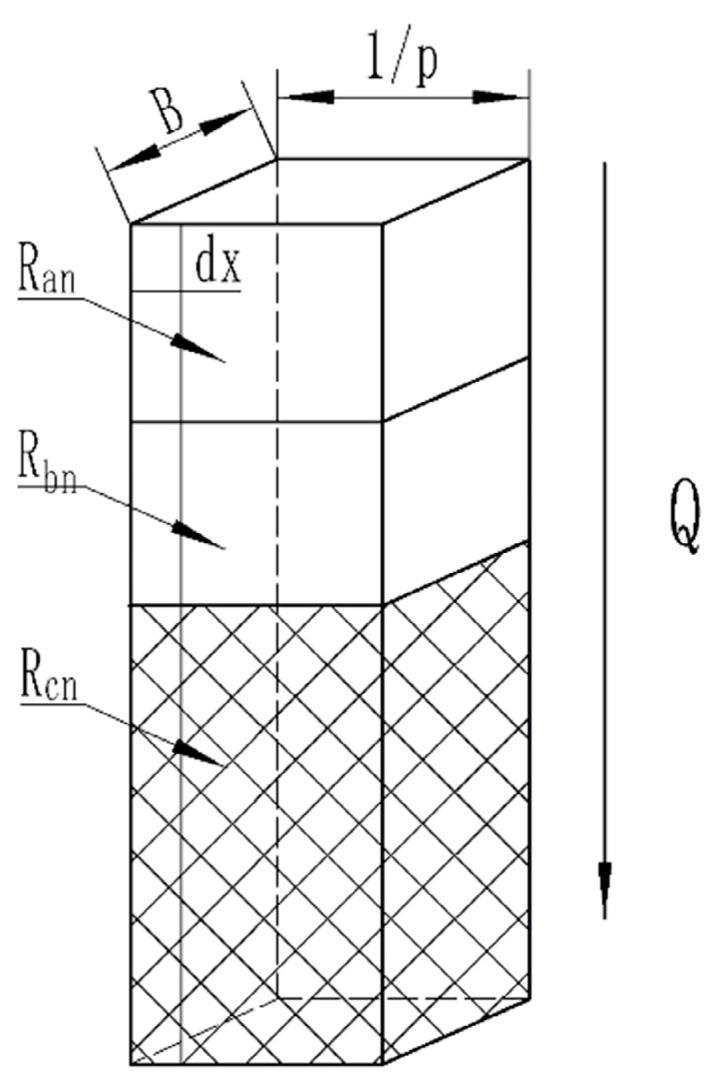
Small ideal unit model.

$R_{o}$ is the total equivalent thermal resistance of the unit, and Figure 5 illustrates the small ideal unit model after equalization.

The total thermal resistance of small ideal units at various positions is assumed to be $R_{n}$; $R_{an}R_{bn}R_{cn}$ is connected in series in accordance with the thermal resistance at various positions. The total thermal resistance $R_{n}$ of the small ideal units is equal to the sum of the resistances of the individual components when connected in series:

(5) $$R_{n}=R_{an}+R_{bn}+R_{cn}$$

The thermal resistance $R_{an}$ of the surface cladding layer is calculated using the Fourier theorem:

(6) $$R_{an}=\frac{h_{a}}{\lambda_{N}\frac{l}{p}}$$

where $\lambda_{N}$ is the thermal conductivity of the surface cladding layer; $h_{a}$ is the surface thickness of the cladding layer; and p is the number of small ideal units.

In order to improve the accuracy of the thermal resistance $R_{bn}\;$ calculation at the junction, an approximate equivalent thickness is obtained by dividing the length of the upper and bottom edges of the function surface by the definite integral. The contact area is calculated based on the length of the Gaussian distribution function in the region. The thermal resistance $R_{bn}$ at the junction can be obtained as follows:

(7) $$R_{bn}=\frac{\frac{\int_{\frac{(n-1)l}{p}}^{\frac{nl}{p}}f\left(x\right)dx}{\frac{l}{p}}}{\lambda_{N}B\cdot \int_{\frac{(n-1)l}{p}}^{\frac{nl}{p}}\sqrt{1+{\left(\frac{df(x)}{dx}\right)}^{2}}dx}$$

where B is the width of the whole composite material, and f(x) is Equation (3).

The calculation of substrate thermal resistance $R_{cn}$ is similar to the bonding thermal resistance. The substrate thickness is calculated by subtracting the thickness of the cladding layer and joint from the overall height:

(8) $$R_{cn}=\frac{H-h_{a}-\frac{\int_{\frac{\left(n-1\right)l}{p}}^{\frac{nl}{p}}f\left(x\right)dx}{\frac{l}{p}}}{\lambda_{c}B\cdot \int_{-\frac{\left(n-1\right)l}{p}}^{\frac{nl}{p}}\sqrt{1+{\left(\frac{df(x)}{dx}\right)}^{2}}dx}$$

where $\lambda_{c}$ is the thermal conductivity of the substrate, and H is the total thickness of the composite element model.

### 3.2. Effective Thermal Conductivity Model of Composite Materials

**Figure 6:**
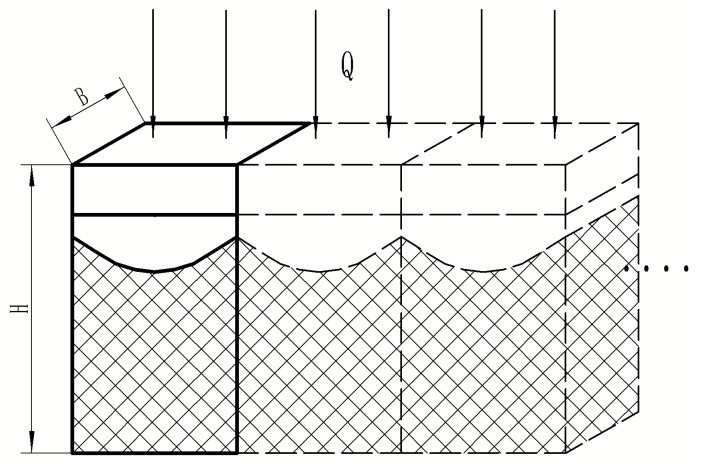
Thermal resistance parallel mode of the effective unit model.

The thermal resistance of the effective element model is obtained using Equations (5)–(8). The physical model of composite materials is composed of multiple element models in parallel, as shown in Figure 6.

We calculate the thermal resistance $R_{o}$ using the element model, and then calculate the actual thermal conductivity according to the actual-size thermal resistance relationship:

(9) $$R_{\alpha }=R_{o}\cdot \frac{2l}{A}$$

$R_{\alpha }$ is the actual thermal resistance of the whole, and the thermal conductivity $\lambda_{\alpha }$ of the composite is calculated according to the Fourier law:

(10) $$\lambda_{\alpha }=\frac{H}{R_{\alpha }AB}$$

That is, according to the above formula, the thermal conductivity model Formula (11) of the composite prepared via laser cladding is finally obtained as follows:

(11) $$\lambda_{\alpha }=\frac{HA\cdot \sum_{n=1}^{p}\left(\frac{\lambda_{N}l}{h_{a}p}+\frac{\lambda_{N}lB\cdot \int_{\frac{(n-1)l}{p}}^{\frac{nl}{p}}\sqrt{1+{\left(\frac{df(x)}{dx}\right)}^{2}}dx}{p\cdot \int_{\frac{(n-1)l}{p}}^{\frac{nl}{p}}f\left(x\right)dx}+\frac{\lambda_{c}B\cdot \int_{-\frac{\left(n-1\right)l}{p}}^{\frac{nl}{p}}\sqrt{1+{\left(\frac{df(x)}{dx}\right)}^{2}}dx}{H-h_{a}-\frac{p\cdot \int_{\frac{\left(n-1\right)l}{p}}^{\frac{nl}{p}}f\left(x\right)dx}{l}}\right)}{ABl}$$

## 4. Verification and Analysis of Heat Conduction Equation

### 4.1. Experimental Preparation Method

The accuracy of the model was assessed by comparing numerical calculations with experimental measurements. The test material used was a pure copper sheet metal measuring 100 mm by 60 mm and having a thickness of 6 mm. The laser cladding preparation equipment used the 1064 nm light source produced by Hans laser (Shenzhen, China), the bc104 coaxial powder feeding cladding device produced by RAYtool (Guangdong, China), the powder feeding device produced by Songxing Welding (Guangzhou, China), and the 4-axis CNC machine tool of Siemens control system (Germany). The process used jp8000 supersonic flame gun produced by Fushan Advanced Surface Technologies Ltd. (Foshan, China) for spraying test.

**Figure 7:**
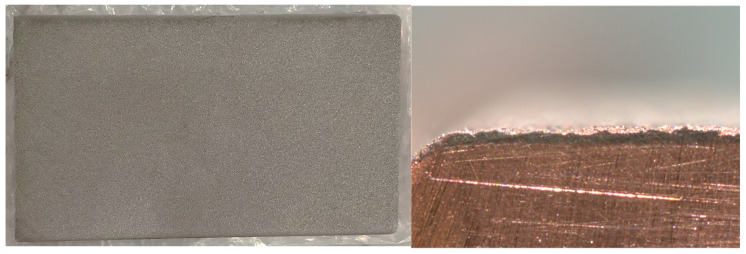
Surface and section of pure copper via supersonic thermal spraying.

The surface of the sample was polished with sandpaper and cleaned with anhydrous ethanol. Ni60A powder (75 μm~48 μm) was selected as the cladding material. Notably, the laser’s absorption rate on the pure copper surface at this frequency is merely 5%, making it challenging to apply a coating to the copper surface [22,23]. The thermal conductivity of copper reaches 401 W/(m×k), making it challenging to concentrate energy and generate a molten pool. Considering the high laser absorption rate of nickel-based materials, we ensured the safety of personnel and equipment. In this investigation, a layer of Ni60A powder with a thickness of 0.3 mm was initially deposited onto the copper plate through supersonic thermal spraying, as depicted in Figure 7 [24]. The process parameters are shown in Table 1.

**Figure 8:**
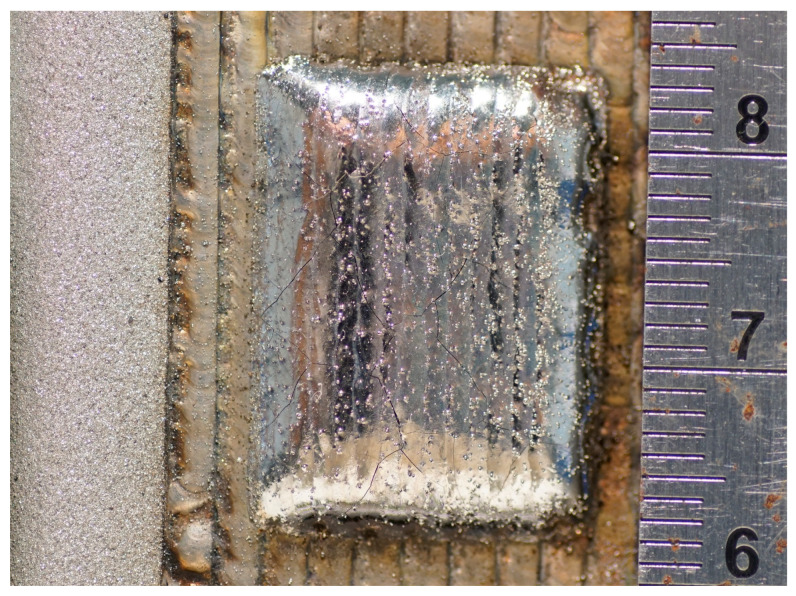
Composite prepared using the above parameters.

The thickness of the Ni60A preset layer is thin, which is influenced by the high thermal conductivity of copper. For the first layer of laser surface cladding, a high-energy concentration is required to generate a molten pool. In the second layer, coaxial powder feeding is employed for laser cladding. Due to the low thermal conductivity of nickel-based materials and the low reflectivity of the laser, molten pools are easily formed, and the power is gradually reduced. The parameters for laser surface alloying and coaxial powder feeding are shown in Table 2. The material surface after multiple stacking according to the above process is shown in Figure 8.

**Figure 9:**
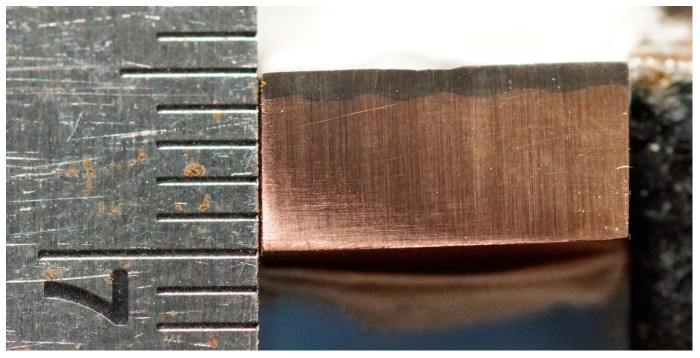
Sample blocks prepared with the above parameters.

The prepared composite material was processed into a size of 10 mm×10 mm×5 mm using a high-precision grinder and wire cutting, with cladding thicknesses of 0.65 mm, 0.75 mm, 0.85 mm, and 0.95 mm, respectively, as shown in Figure 9.

**Figure 10:**
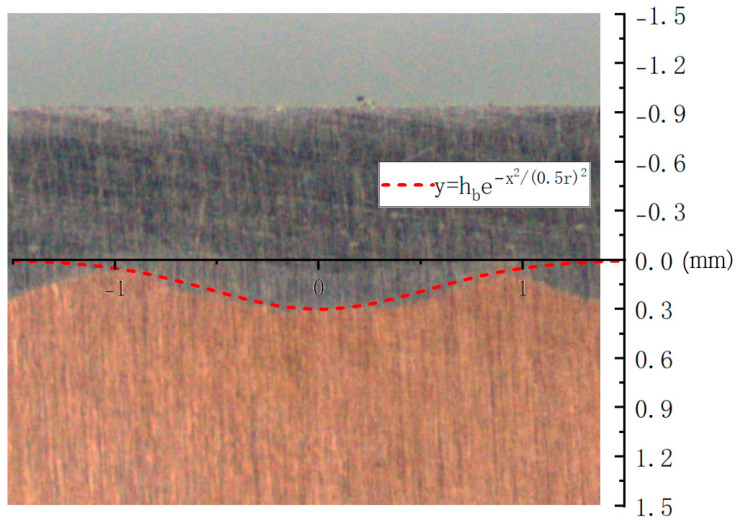
Composite cross section overlapped with Equation (12).

The composite cross-section image was taken using a macro camera, the image of Equation (12) was drawn using a software, and the two images were overlapped. As shown in Figure 10, the interface between the surface coating and the substrate is Gaussian distribution and highly fitted, which has a strong correlation. The correlation between the unit model is also proven, where

(12) $$f\left(x\right)=h_{b}\exp\left(-x^{2}/{\left(0.5r\right)}^{2}\right)$$

According to the ASTM El461-01 standard [25], the thermal diffusivity of the composite was measured using a laser thermal conductivity meter (LAF427) at different temperatures, and then the sample density was measured using the drainage method. Finally, the thermal conductivity was calculated by using the following formula:

(13) $$\lambda =\alpha \times \rho \times C_{\rho }$$

where α is the thermal diffusion coefficient; ρ is the density; and $C_{\rho }$ is the specific heat capacity.

**Figure 11:**
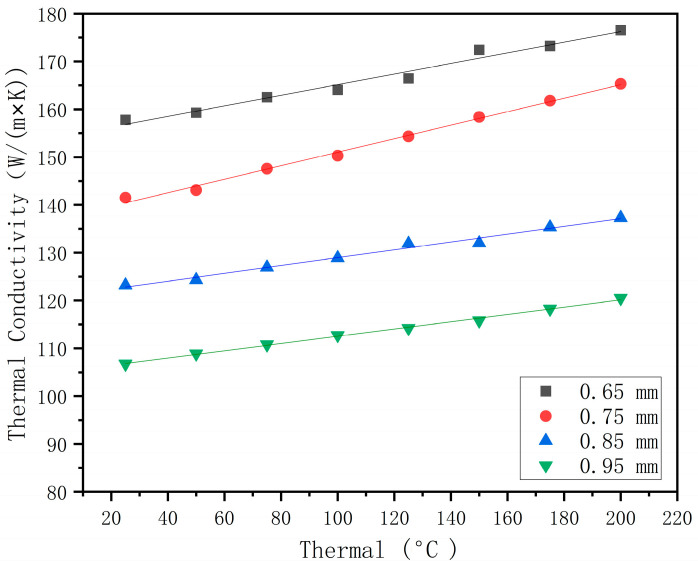
Experimental thermal conductivity of composite at different temperatures.

The experimental thermal conductivity was obtained according to the above formula, as shown in Figure 11. According to the results, it can be seen that the effective thermal conductivity of the composite material is 25 W/(m×K) compared with that of the general material die steel H13. Through the establishment of the model, it can be found that the thermal conductivity decreases with an increase in the coating, and the approximate value range of the cladding layer thickness can be determined.

### 4.2. Model Comparison and Analysis

According to the preparation process, the following prediction and calculation parameters were selected: the radius of the laser beam is 1.5 mm, the distance between two adjacent laser cladding layers is 2 mm, the height of the first laser cladding layer is 0.3 mm, and the number of small ideal units is 100. It is verified according to the experimental material parameters that the cladding layer uses powder metal containing the content shown in Table 3.

**Figure 12:**
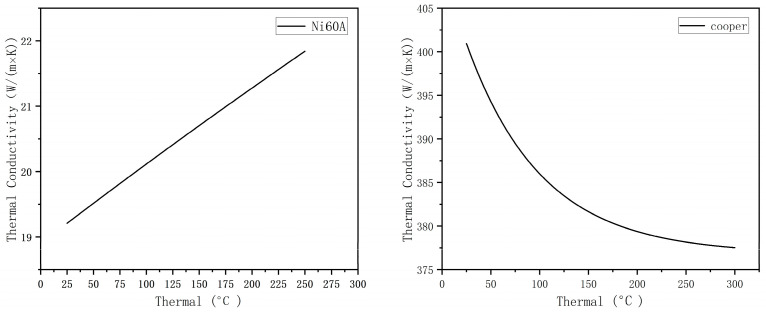
Thermal conductivity of Ni60A and copper at different temperatures.

The thermal conductivity of Ni60A material was derived using JMatPro 6.0 (Sente Software Ltd., UK) software, and the thermal conductivity is shown in Figure 12.

**Figure 13:**
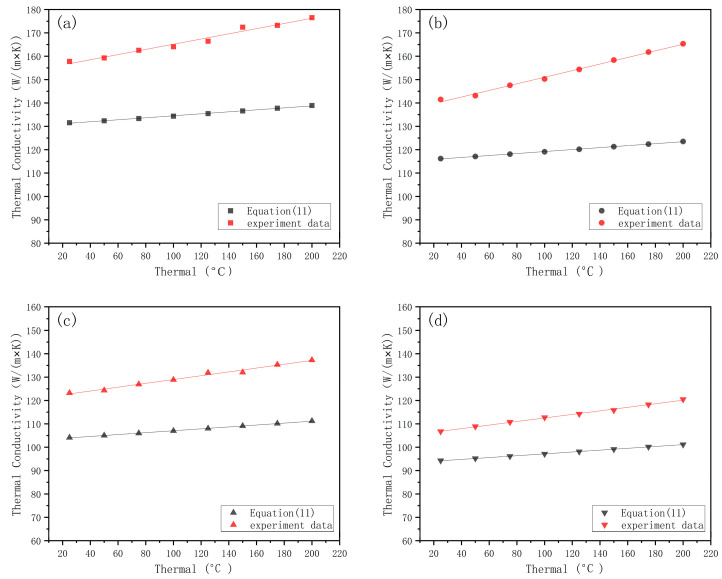
Predicted and measured values of effective thermal conductivity at different temperatures. Effective thermal conductivity of the cladding layer with thicknesses of (**a**) 0.65 mm, (**b**) 0.75 mm, (**c**) 0.85 mm, and (**d**) 0.95 mm.

The model’s predicted values were compared with the experimental measured values using Equation (11). As shown in Figure 13, the effective thermal conductivity of different cladding layer thicknesses increases with temperature. The overall average accuracy of the predicted results of the mathematical model is 82.14%, and the overall trend is in good agreement with the experimental data. The accuracy increases with an increase in coating thickness.

**Figure 14:**
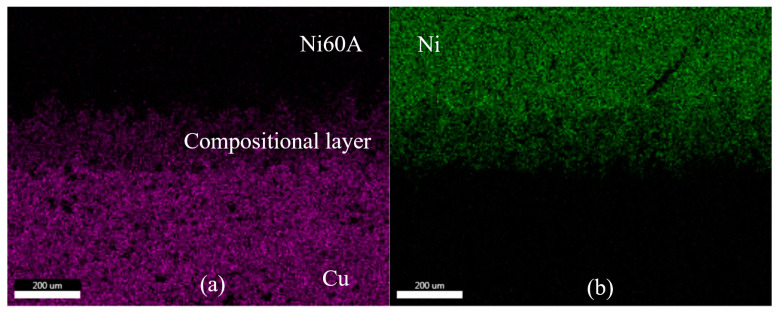
Image obtained when scanning the junction using energy-dispersive spectrometer: (**a**) Cu to (**b**) Ni through the interfaces.

The model error analysis shows that the overall mathematical model has a lower deviation, and the error increases with a decrease in coating thickness. The main reason for the lower deviation is that the thermal conductivity of the material used in the model is lower than the actual situation. To ensure its universality, the two models ignore the alloy produced by combining two dissimilar alloy materials in the laser cladding process. In reality, the energy-dispersive spectrometer detects the joint of the two materials, as shown in Figure 14. There is a part of metallurgical bonding at the junction. According to the research conducted by Bai et al. [26], the thermal conductivity between the Ni60A powder and the copper substrate increases with an increase in copper content and temperature. When the copper content was 30%, the composite showed a higher thermal conductivity (26.8 W/(m×K)) than the thermal conductivity of Ni60A set in the prediction model. Therefore, the overall prediction value of the mathematical model has lower deviation, and the error increases with a reduction in the thickness of the cladding layer.

To study its influence, the thermal conductivity of Cu/Ni alloy is introduced into the model Formula (11). The thermal conductivity of the composite material is calculated as follows:

(14) $$\lambda_{\alpha }=\frac{HA\cdot \sum_{n=1}^{p}\left(\frac{\lambda_{N}l}{h_{a}p}+\frac{\lambda_{c\_n}lB\cdot \int_{\frac{(n-1)l}{p}}^{\frac{nl}{p}}\sqrt{1+{\left(\frac{df(x)}{dx}\right)}^{2}}dx}{p\cdot \int_{\frac{(n-1)l}{p}}^{\frac{nl}{p}}f\left(x\right)dx}+\frac{\lambda_{c}B\cdot \int_{-\frac{\left(n-1\right)l}{p}}^{\frac{nl}{p}}\sqrt{1+{\left(\frac{df(x)}{dx}\right)}^{2}}dx}{H-h_{a}-\frac{p\cdot \int_{\frac{\left(n-1\right)l}{p}}^{\frac{nl}{p}}f\left(x\right)dx}{l}}\right)}{ABl}$$

where $\lambda_{c\_n}$ is the thermal conductivity of Cu/Ni alloy.

**Figure 15:**
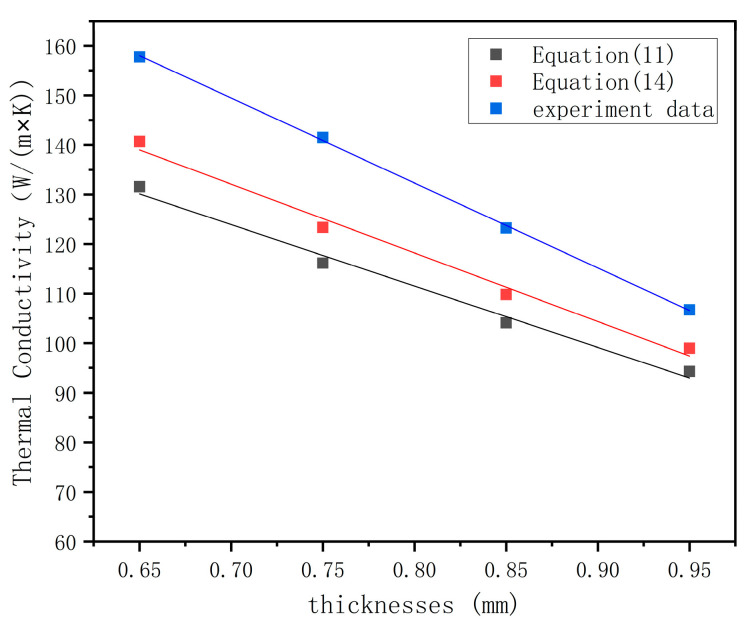
Model comparison after considering the thermal conductivity of the transition layer.

According to the thermal conductivity (26.8 W/(m×K)) in [26], the calculated results of Formula (14) were compared with the data in Figure 13. The comparison of the results is shown in Figure 15. The accuracy of the overall model (Formula (15)) improved by 4.97% after considering metallurgical integration through data comparison, as shown in Figure 15.

Through the above analysis, it can be found that the systematic error comes from underestimating the thermal conductivity of the sediment. The main impact is in the transition-layer boundary, the size and element distribution of which are affected by multiple factors K. Halmesova et al. [14] found that the use of different process parameters during laser cladding preparation resulted in different metallurgical bonding areas between the two materials, which affected the thermal conductivity. The metallurgical bonding areas under different parameters need to be retested and calculated. Bonny Onuike et al. [2] found that the use of laser cladding to produce materials resulted in a finer grain structure compared to traditional production techniques, resulting in different thermal conductivity coefficients for the same material. In order to improve the accuracy of the model, it is necessary to measure the thermal conductivity of each part of the composite material preparation process to improve its accuracy. This is clearly contrary to the purpose of establishing a model to improve the design speed of composite materials. To ensure that the model has some guiding significance for material design, this study simplifies the transition boundary to a certain extent.

**Figure 16:**
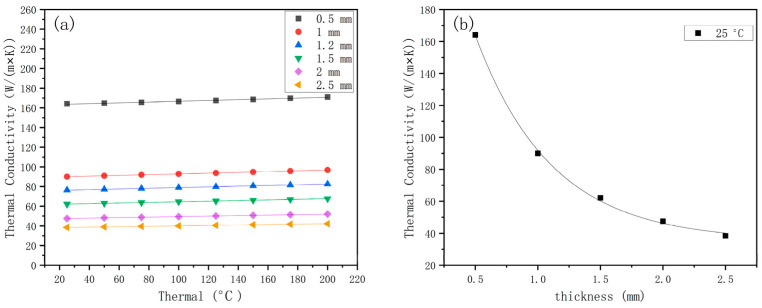
Effective thermal conductivity under different thicknesses. (**a**) Thermal conductivity of composites with different thicknesses (**b**) Thermal conductivity of composites of different thicknesses at 25 °C.

Through a comparison of the model established in this study, it was found that the relationship between thermal conductivity and key parameters varied positively with changes in key parameters. The model can be used to roughly predict thermal conductivity under different parameters. At the same time, the key parameters of composite materials can be designed based on actual needs. Figure 16 shows that the effective thermal conductivity decreases by 75% logarithmically as the thickness of the surface cladding layer increases from 0.5 mm to 2.5 mm. More specifically, when the spot diameter is 3 mm and the overlap rate is 33.3%, the effective thermal conductivity decreases logarithmically, with the thickness increasing from 0.5 mm to 2.5 mm. This factor has a strong correlation, which should be considered first in future research on materials’ thermodynamic and mechanical properties.

The proposed mathematical model can easily obtain the thermal conductivity of composites prepared using different cladding materials and processes. It provides theoretical and practical guidance for future research on the thermodynamic properties of composites prepared via laser cladding.

## 5. Conclusions

In this study, a mathematical model was proposed to predict the effective thermal conductivity of laser-clad nickel-based alloy composites on copper surfaces. The model was verified through comparison and experimental data. From the results obtained, we draw the following conclusions:

- By comparing the experimental data, a mathematical model (Equation (11)) was established to predict the effective thermal conductivity, with an accuracy of 82.13%, and the overall trend was in good agreement with the experimental data.
- By comparing the experimental data, the accuracy of the prediction results increases with an increase in the cladding layer thickness, and the overall deviation of the mathematical model results is found. The reason for the overall error is that to ensure its universality, the model ignores the alloy produced by the combination of two different alloy materials in the process of laser cladding. In fact, the metallurgical bonding of Ni/Cu at the joint improves the thermal conductivity of the material. When the thickness of the cladding layer is thin, the effect of metallurgical bonding should be considered.

To sum up, these key parameters not only affect the quality of the coating but also affect the effective thermal conductivity of composites. Therefore, establishing this mathematical model can provide theoretical guidance and optimization direction for the study of materials and the future parameter selection of composite materials.

## Figures and Tables

**Table 1:** Process parameters of HVOF on copper surface.

Technique	Powder Drying Temperature (°C)	Kerosene Flow Rate (L/h)	Oxygen Flow Rate (L/min)	Nitrogen Flow Rate (L/min)	Powder Feed Rate (L/min)	Spray Distance (mm)	Linear Torch Velocity (mm/s)
HVOF	300	19	750	14	65	310	600

**Table 2:** Laser surface alloying and laser cladding process parameters.

Layers	Process Method	Power (kW)	Speed (mm/s)	Laser Beam Radius (mm)	Adjacent Distance (mm)	Shielding Gas Ar Flow (L/min)	Powder Feed Rate (RPM)
1	Laser surface alloying	3000	7	1.5	2	12	0
2	Laser cladding	1500	5	1	1	12	50
3	Laser cladding	1400	5	1	1	12	50
4	Laser cladding	1000	5	1	1	12	50
5	Laser cladding	1000	5	1	1	12	50

**Table 3:** Content of Ni60A powder.

Chemical Composition (wt.%)						
Material name	Ni	C	Si	B	Cr	Fe
Ni60A	Bai	0.9	4	3.2	16	5

## Data Availability

The data used to support the findings of this study are included within the article.
